# Strain softening of nano-scale fuzzy interfaces causes Mullins effect in thermoplastic polyurethane

**DOI:** 10.1038/s41598-017-00904-3

**Published:** 2017-04-20

**Authors:** T. Sui, E. Salvati, S. Ying, G. Sun, I. P. Dolbnya, K. Dragnevski, C. Prisacariu, A. M. Korsunsky

**Affiliations:** 1 MBLEM, Department of Engineering Science, University of Oxford, Parks Road, Oxford, OX1 3PJ UK; 2 Diamond Light Source, Harwell Campus, Didcot, OX11 0DE UK; 3 Institute of Macromolecular Chemistry “Petru Poni”, Aleea Grigore Ghica Voda, Nr. 41A, Iasi, 700487 Romania; 4 State Key Laboratory of Geomechanics and Geotechnical Engineering, Institute of Rock and Soil Mechanics, Chinese Academy of Sciences, Wuhan, Hubei 430071 China

## Abstract

The strain-induced softening of thermoplastic polyurethane elastomers (TPUs), known as the Mullins effect, arises from their multi-phase structure. We used the combination of small- and wide- angle X-ray scattering (SAXS/WAXS) during *in situ* repeated tensile loading to elucidate the relationship between molecular architecture, nano-strain, and macro-scale mechanical properties. Insights obtained from our analysis highlight the importance of the ‘fuzzy interface’ between the hard and soft regions that governs the structure evolution at nanometre length scales and leads to macroscopic stiffness reduction. We propose a hierarchical Eshelby inclusion model of phase interaction mediated by the ‘fuzzy interface’ that accommodates the nano-strain gradient between hard and soft regions and undergoes tension-induced softening, causing the Mullins effect that becomes apparent in TPUs even at moderate tensile strains.

## Introduction

Thermoplastic polyurethane (TPU) elastomers are versatile polymer materials that find numerous applications due to the outstanding combination of thermal and mechanical properties. TPUs are block co-polymers that have the morphology of nano-composites consisting of hard regions embedded within contiguous soft regions, with nano-scale gradient transitions between them that are structurally accommodated by the “fuzzy interface” regions^1, 2^. Due to this intricate hierarchical architecture and the presence of fuzzy interfaces, the internal strain within these materials depends on the length-scale of consideration, i.e. macroscopic strain is different from the nano-scale strain, which in turn is different from the small-scale interatomic strain^3^. One interesting aspect of the mechanical behaviour of these materials is that they exhibit the so-called **Mullins effect** ^4^. The term “Mullins effect” refers to the phenomenon of apparent softening (the reduction of macroscopic tangent modulus) under repeated tensile loading, and has attracted a great deal of attention from researchers for more than six decades, with extensive experimental evidence obtained for filled and crystallized rubbers, such as natural rubbers (NR)^5, 6^, styrene butadiene rubber (SBR)^7, 8^, and for many other rubber-like materials, including TPUs^9, 10^. However, although various models proposed allow matching the macroscopic behaviour, none so far provided a satisfactory structure-based explanation of this effect that remains an elusive challenge^11^.

A variety of experimental techniques have been utilized to investigate and characterize the deformation behavior of TPUs^12–15^. However, most studies use macroscopic deformation response of these materials as a basis for making hypotheses about the nature of deformation at finer scales. A very limited amount of effort has been devoted to direct exploration of deformation at the micro- and nano-scales, and understanding of the link between the multi-phase material architecture and strain inhomogeneity that it induces across the scales. *In situ* multiple length scale deformation analysis using multi-modal synchrotron X-ray techniques allows revealing the intricate links between strain and structure across the scales.

In our previous study synchrotron-based Small Angle X-ray Scattering (SAXS) has been demonstrated to be sensitive to the presence and deformation of the **fuzzy interface**, and capable of obtaining quantitative nano-scale structural information during *in situ* deformation of TPUs^16, 17^. In addition, synchrotron-based Wide Angle X-ray Scattering (WAXS) can be used to quantify the material’s crystallographic properties and the relationship between atomic scale internal strain and external load. Although the combined SAXS/WAXS technique has become popular for evaluating the structural evolution of polyurethane and other elastomers^18–20^, it is often used for sample characterization before and after deformation, or at best during a single monotonic loading experiment, rather than in the course of repeated loading. The motivation of the present investigation is to address the clear need to understand the processes that unfold during repeated loading of TPU’s. We tackle this challenge by conducting a combined systematic experimental and modelling study of the structure-mechanical relationship of TPU’s. This allows us to elucidate the physical mechanisms of cyclic strain softening under repeated loading. In this study we explore the evolution of the hierarchical architecture of a polyurethane at the macro-, nano- and atomic scales during repeated uniaxial tensile straining using simultaneous *in situ* SAXS/WAXS techniques, thus obtaining crucially needed observational basis for improved insight into the physical origins of the Mullins effect.

The existing physical interpretations of the Mullins effect are linked to microstructural changes and/or damage. However, due to the lack of suitably mature experimental methods for multiple scale structural evolution analysis, most approaches have had no validation at the nano-structural stress level, or have relied on unproven assumptions. Consequently, none of the proposed theories have received proper experimental validation. In fact, although some of these models can fit the macroscopically observed stress-softening phenomenon in rubber-like materials, a great deal of discrepancy exists between the values of physical parameters used to achieve agreement^21–26^.

Based on *in situ* multi-scale strain analysis in a TPU, the present work proposes a double inclusion model to describe the interactions between phases and length scales, and to interpret multi-scale experimental data from a physical perspective. This study demonstrates that the softening mechanism responsible for the reduction of macroscopic stiffness under repeated loading is driven by the nature of multi-phase hierarchical structure of polyurethane, and in particular by the existence and structural changes in the “fuzzy interface”.

## Results

### Multiple length-scale strain analysis

The combined synchrotron X-ray experimental setup for simultaneous WAXS, SAXS and imaging was described in our previous publication^3^. For the present experimental study, several additional beamline modifications were implemented, including: (i) the use of Multilayer Monochromator (MLM) (Ru/B4C) that allows using higher X-ray flux in the range 8–20 keV, and results in better quality scattering patterns; (ii) wider *q* range coverage achieved by increasing the sample-to-detector distance to improve SAXS data acquisition; (iii) shorter gauge length dog-bone sample, designed and used in combination with a smaller and more accurate loading rig, enabling the larger strain range to be achieved in the sample. As a consequences of all these improvements, the manifestation of Mullins effect becomes more prominent.

The sample was subjected to multiple cycles of uniaxial incremental tensile loading and unloading using a miniature test rig (Deben, UK), which recorded the macroscopic deformation parameters of the sample. Five maximum load values were chosen, namely, 4.5N, 6.5N, 8N, 9.5N and 10N. Measurements were conducted at several points during each loading and unloading step.

**Figure 1:**
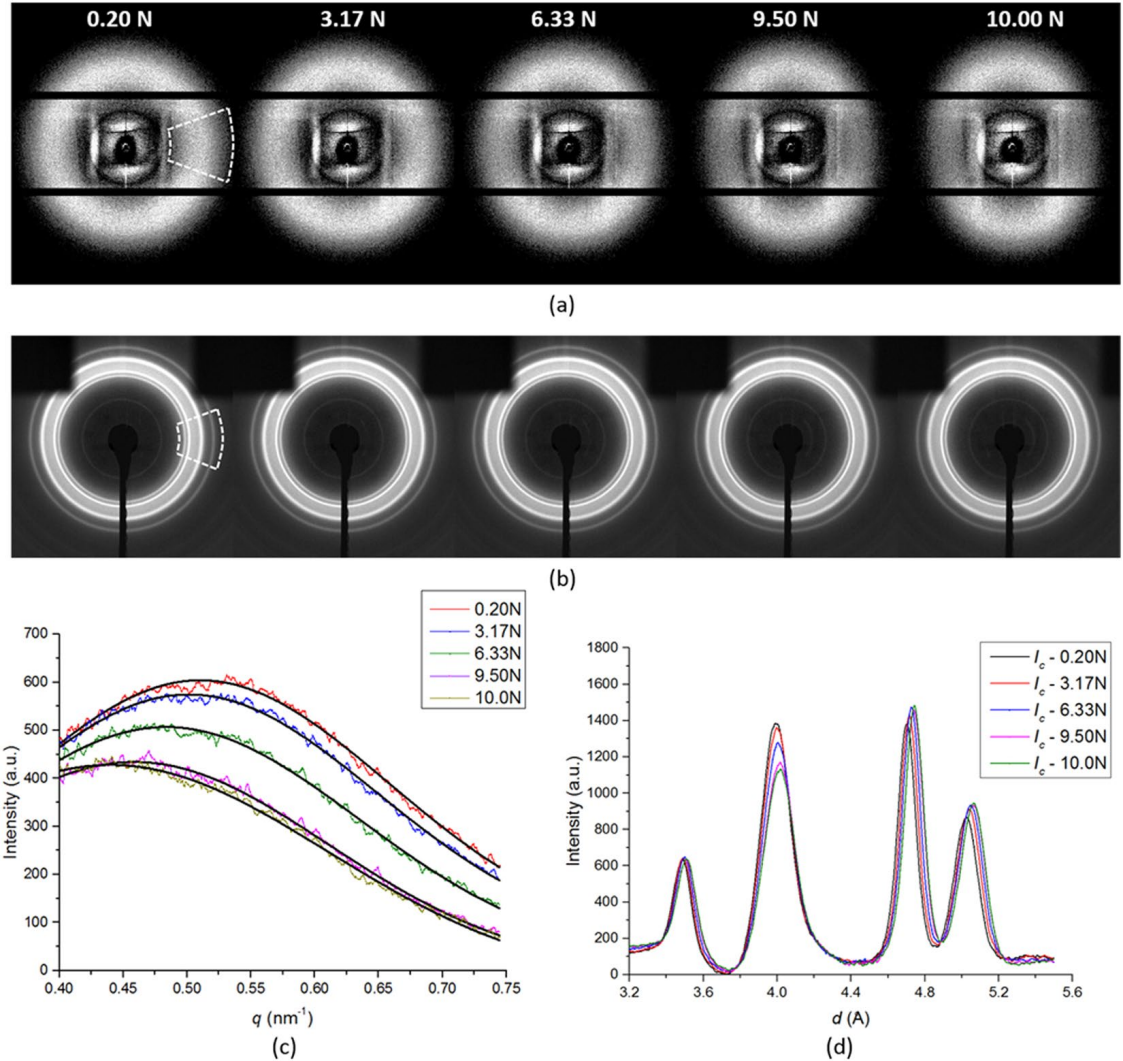
Structural evolution of 2D scattering patterns and 1-D profiles. Selected **(a)** SAXS patterns and **(b)** WAXS patterns for final loading stage (0.2–10 N); the family of equivalent 1D profiles of scattered **(c)** SAXS intensity with Gaussian curve fitting and **(d)** WAXS intensity with Gaussian curve fitting.

An illustration of the results is given in terms of 2D SAXS and WAXS patterns of the final cycle (0.2–10N) in Fig. 1a,b. The evolution of the SAXS patterns is presented in Fig. 1a that shows the gradual change from circular to elliptical shape. No obvious change can be seen directly in the corresponding WAXS patterns in Fig. 1b. In order to extract the information about structural changes reflected in the 2D SAXS and WAXS patterns, and for the purpose of quantitative analysis of these patterns, 1D radial binning was conducted for each pattern along the loading *x*-axis indicated by the dashed segment in Fig. 1a,b. The results are shown in Fig. 1c,d. From the 1D radial line profiles of SAXS patterns it is observed in Fig. 1c that the magnitude of the scattering vector q* corresponding to the maximum scattered intensity decreases with increasing load, whilst in the 1D WAXS patterns after the deduction of amorphous background (see Methods section), the peak center shift is observed for each of the four crystalline peaks. It is noted that the peaks follow different trends in response to the increasing load, revealing intergranular interaction within the crystalline fraction of the polymer. Precise strain values were obtained by determining peak center positions using Gaussian fitting, both for SAXS radial profiles in Fig. 1c and for individual WAXS peaks in Fig. 1d. This has been conducted for the entire load history described above.

**Figure 2:**
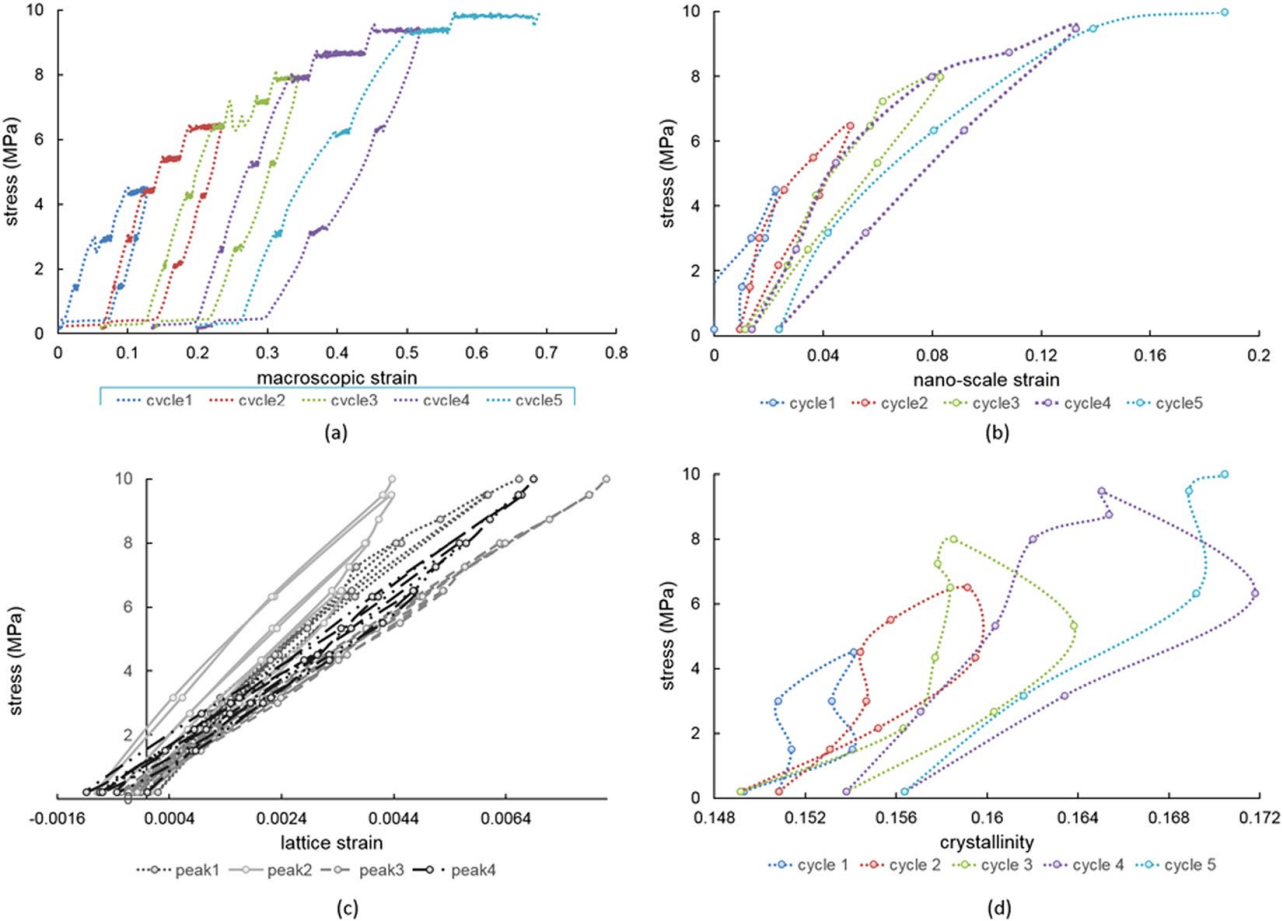
Multi-scale strains visualization. **(a)** Macroscopic strain evolution registered by Deben loading rig; **(b)** Nano-scale strain evolution interpreted from SAXS patterns along loading direction; **(c)** Atomic scale strain evolution derived from four diffraction peaks of WAXS patterns; **(d)** Crystallinity evolution obtained from WAXS patterns.

The evolution of strains at multiple length scales over the entire loading history is presented in Fig. 2a–c, respectively, that includes the macroscopic strain measured by the Deben rig, nano-scale strain calculated from the SAXS patterns, and the atomic strain calculated from the WAXS patterns. In addition, the evolution of the degree of crystallinity calculated from each WAXS pattern is shown in Fig. 2d.

The results reveal that there exists a general correlation between strains perceived at different scales. Namely, at each load the overall strain ratio between macro-scale strains to nano-scale strains to atomic level strains is close to ~100: 30: 1. The softening phenomenon reflected in the decrease in the stress-strain slope after each cycle is apparent from Fig. 2a,b at the macro- and nano- scales. No obvious change in the stress-strain slope can be identified at the atomic scale between different cycles from Fig. 2c.

It is worth noting that following load application and removal, increasing tensile residual strains appear at the macro- and nano- scales, whilst increasing compressive residual strains appear at the atomic scale. This phenomenon is well documented for this family of polymers which exhibit hysteretic strain evolution and in which increasing tensile residual strain is accumulated at the macroscopic scale. Although this is often attributed to the large stiffness and strength mismatch between the hard and soft regions within the material, only phenomenological explanations have been provided so far that are not based on structure considerations.

To fill this gap, we make direct observation and analysis of deformation at the atomic and nano-scale. WAXS results allow strain evaluation at the atomic scale from the crystalline regions within the material (Fig. 2c). Not only do individual reflections exhibit different slope of elastic response to the applied load, but each of the peaks displays different amount of hysteretic behaviour. This phenomenon is well-documented in polycrystalline metallic alloys^27^ possessing elastic and plastic anisotropy. These results indicate that the chain folding arrangement within the hard region due to physical cross-linking must be similar to a polycrystalline aggregate with anisotropy of structure and properties that manifests itself in the apparent stiffness dependence on orientation.

Finally, we note that crystallinity increases gradually within each loading cycle, and exhibits a partially reversible trend, although no significant change is observed in the slope of the graph.

**Figure 3:**
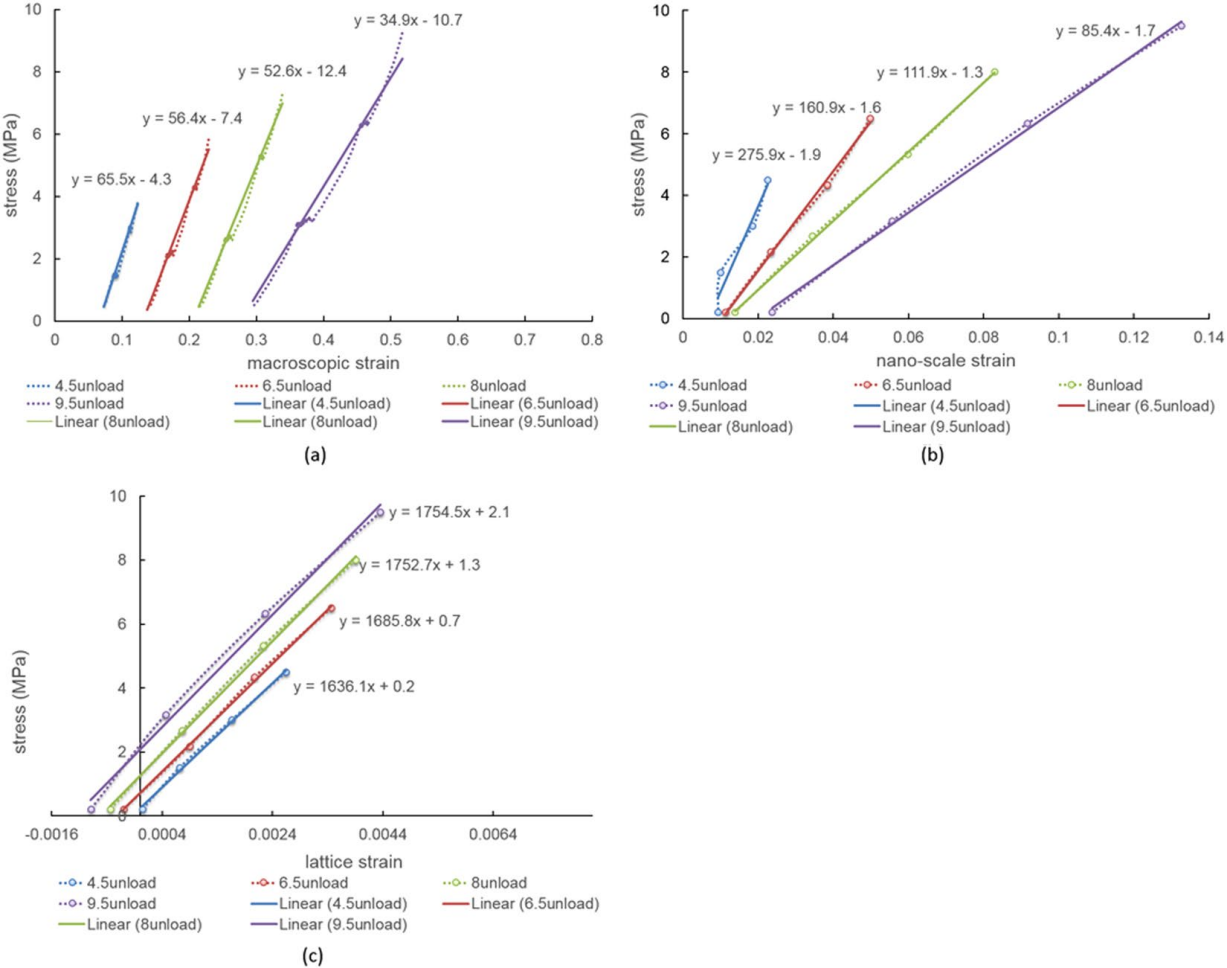
Softening behavior during cycles at different length scales. Macroscopic strain evolution of **(a)** unloading for each cycle; nano-scale strain evolution of **(b)** unloading for each cycle; atomic strain evolution (from peak 2) of **(c)** unloading for each cycle.

For the purpose of quantitative analysis we use the “apparent modulus” (*K*) that corresponds to the slope of the graphs, and is defined as the ratio of the increment of applied stress to that of strain at the appropriate scale. Figure 3 shows apparent modulus changes with linear fitting at each cycle during unloading. Gradual decrease of the apparent modulus (from 65.7 MPa to 34.9 MPa) can be seen at the macro-scale from cycle to cycle in Fig. 3a. At the nano-scale, the apparent modulus decreases from 156.2 MPa to 58.2 MPa between successive unloading steps, as shown in Fig. 3b. Comparing the unloading slopes in the last and initial cycles it is found that the degree of softening at the nano-scale (SAXS apparent modulus drop of ~70%) is greater than that at macro-scale (apparent modulus drops of ~50%). In terms of the atomic level strain, the apparent modulus of the most prominent peak 2 selected for analysis (Fig. 3) is found to increase slightly from 1626.2 MPa to 1831.5 MPa in successive unloading steps. In order to make sense of these multi-scale observations of strain evolution we employ a hierarchical model described below. In the model, we made the approximation that the apparent modulus *K*_*saxs*_ (based on the analysis of SAXS results) is equal to *K*_*fi*_, i.e. the apparent modulus of the fuzzy interface. This is justified by the sensitivity of strain value obtained from SAXS pattern analysis to the fuzzy interface that was found and described in our previous work^3^.

### Double-inclusion Eshelby model

**Figure 4:**
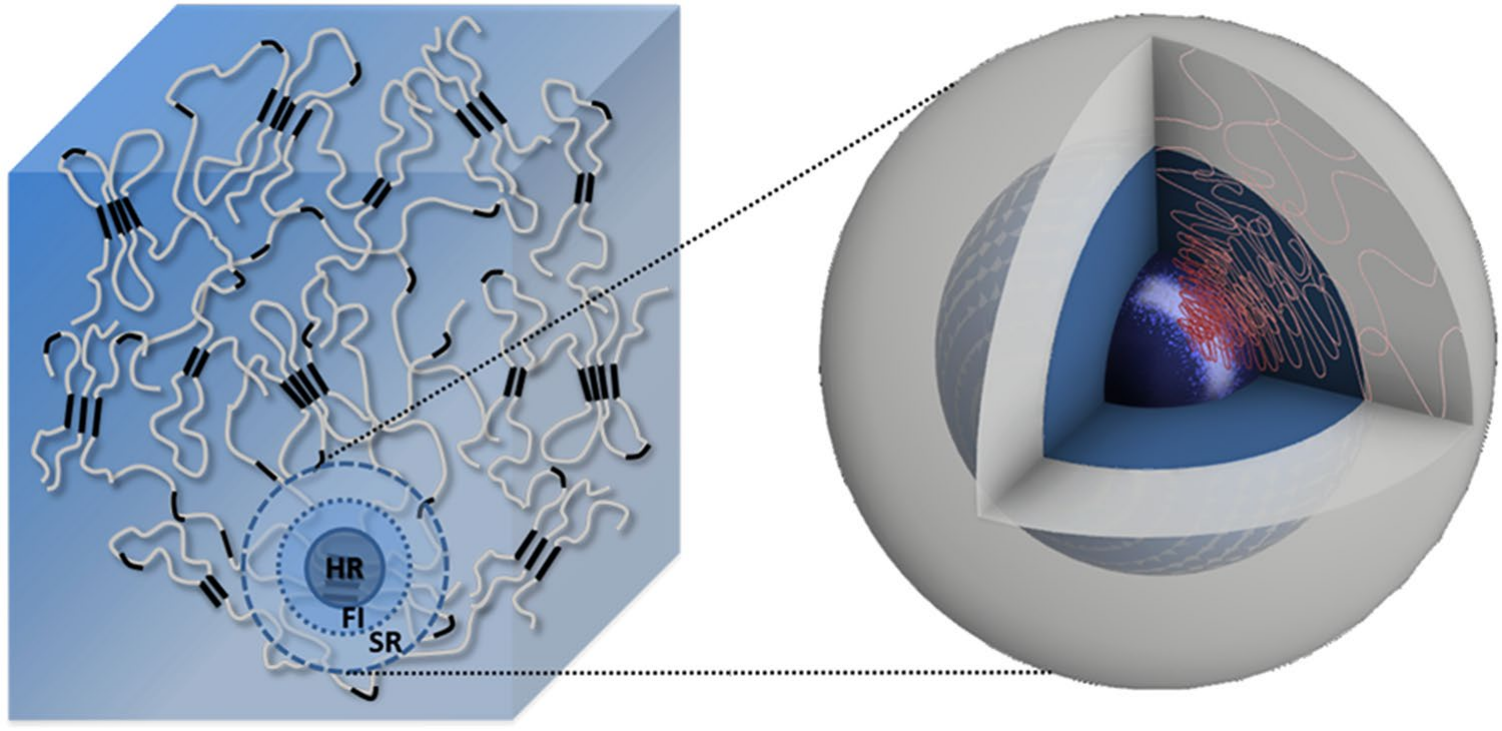
Illustrations of TPU representative volume element and the double-inclusion model. TPU is thought of as consisting of hard regions (HR) embedded within contiguous soft region (SR) matrix, with gradient transitions between HR and SR that are structurally accommodated by the “fuzzy interface” (FI). In the double-inclusion model, at level 1, the inclusions represent the combination of HR & FI, and SR serves as the matrix. At level 2, the inclusion represents only the HR, and FI serves as the matrix.

In this section we describe the construction of a compact model capable of matching the experimentally observed stress-strain response at all structural levels. We concentrate our attention on the material response during unloading, as it is largely elastic and allows reliable, statistically robust evaluation of apparent moduli. Model matching to each unloading stage then allows to identify the consequences of the inelastic processes that occur during loading, revealing the interaction between phases at different length scales, changes in the volume fraction and stiffness of phases, etc. The proposed double-inclusion Eshelby model is described in more detail in the Methods section. As model input we use the elastic moduli (*E*) and volume fractions (*f*) of individual phases. We introduce three distinct phases as illustrated in Fig. 4. The macroscopic model corresponds to the cuboidal representative volume element shown in blue that consists of hard region (HR), fuzzy interface (FI) and soft region (SR). These regions, in turn, at the finer level are all composed of hard segments (HS) and soft segments (SS) characteristic for polyurethane molecular structure that are shown in black and white, respectively. HR refers to the hard regions characterized by high density, high degree of physical cross-linking and regularity of chain arrangement (crystallinity), and high stiffness. SR denotes the soft regions where physical cross-linking is not present or is very weak, and both the density and stiffness are low. FI denotes the fuzzy interface phase that possesses intermediate properties between HR and SR, and corresponds to the regions of gradual transition in the morphology (e.g. degree of physical cross-linking) and properties (density, stiffness) between the two extremes. This is the most interesting region for study, since it accommodates the very large strain difference (up to a factor of ~100) between hard and soft regions within the material, and, as we show below, undergoes most change under even moderate tensile straining and is responsible for the macroscopically observed Mullins effect. Our ability to interrogate the response of this important region stems from the close connection between the density and deformation of the FI, on the one hand, and the SAXS pattern, on the other^3^.

Since we probe the structure and behaviour of TPU using X-ray scattering and imaging methods, it is logical to choose electron density as the primary basis for discrimination between phases. In the model, the initial volume fraction of the hard region HR is chosen to be *f*_*hr*_ = 0.33, in line with the information available from the literature^28, 29^. To assist model interpretation, we fix the volume fraction of the FI at *f*_*fi*_ = 0.1. This assumption is convenient from a number of viewpoints. Firstly, by the very nature of its definition, the FI is identified somewhat vaguely, and does not allow precise quantification. Furthermore, fixing the notional volume fraction of FI allows focusing our attention on the property evolution of this phase, notably its stiffness, which in turn can be correlated with morphological changes, damage, etc. In contrast, the volume fractions of HR and SR are allowed to evolve as a consequence of deformation, with that of SR determined by the balance equation *f*_*sr*_ = 1 − *f*_*hr*_ − *f*_*fi*_. To achieve good agreement with the experimentally observed apparent moduli *K*(exp) (*K*_*mac*_, *K*_*hard*_ and *K*_*saxs*_ */K*_*fi*_), model inputs were refined, including the three elastic moduli *E*(mod) (*E*_*hard*_, *E*_*fi*_ and *E*_*soft*_) and the variable volume fractions *f*_*hr*_ and *f*_*sr*_ for different cycles. The obtained results are summarized in Table 1. As a consequence of model refinement, the values of the apparent modulus *K*_*soft*_ were also obtained, and are presented in the Table.

**Table 1:** Changes of the volume fraction, apparent modulus and elastic modulus of different phases in unload cycles.

	cycle 1	cycle 2	cycle 3	cycle 4
**Volume fraction**				
*f*_*hr*_	0.33	0.27	0.25	—
*f*_*fi*_	0.1	0.1	0.1	—
*f*_*sr*_	0.57	0.63	0.65	—
**Apparent Modulus (MPa)**				
*K*_*mac*_ (*exp*)	65.5	56.4	52.6	34.9
*K*_*hard*_ (*exp*)	1636.1	1685.8	1752.7	1754.5
*K*_*SAXS*_/*K*_*fi*_ (*exp*)	275.9	160.9	111.9	85.4
*K*_*soft*_ (*mod*)	38.8	37.2	36.2	—
**Elastic Modulus (MPa)**				
*E*_*hard*_ (*mod*)	2470.3	2693.5	2846.9	—
*E*_*fi*_ (*mod*)	237.3	141.1	98.2	—
*E*_*soft*_ (*mod*)	28.3	28.4	28.2	—

*f*_*hr*_ *f*_*fi*_ and *f*_*sr*_ refer to the volume fractions of the hard region (HR), fuzzy interface (FI) and soft region (SR); *K*_*mac*_, *K*_*hard*_ and *K*_*saxs*_ (or *K*_*fi*_) are experimentally measured (*exp*) macro-, atomic scale and nano-scale apparent moduli. *K*_*soft*_ is the apparent modulus obtained from the refined model (*mod*) for pure soft region; *E*_*hard*_, *E*_*fi*_ and *E*_*soft*_ are the refined (*mod*) elastic moduli for each phase.

The evolution of the apparent moduli and the elastic moduli of different phases in successive unloading cycles reveals the nature of the underlying multi-phase interactions. For the first three cycles (cycles 1–3 from Table 1), it can be seen that the volume fraction of the SR increases gradually from 0.57 to 0.65 at the expense of the HR volume fraction, which decreases from 0.33 to 0.25. The elastic modulus of the soft region remains almost constant (~28 MPa), while that of the hard region increases slightly with each cycle, from 2470.3 MPa to 2846.9 MPa. The striking conclusion is that the elastic modulus of the fuzzy interface (*E*_*fi*_) shows the most dramatic change from 237.3 MPa to 98.2 MPa between the first and third cycle. Remarkably, this drastic reduction occurs already at the moderate strains (<50%). This indicates that the origins of the macroscopically observed softening phenomenon (Mullins effect) must be primarily associated with the modification (rearrangement and softening) within the fuzzy interface. In contrast, the changes that occur within the material at the larger strain attained in cycle 4 are likely to be associated with more extensive structural changes, e.g. with damage affecting SR to a greater extent. This is consistent with the literature reports of the onset of damage in single phase soft amorphous polymers occurring at large stretch ratios^30^.

## Discussion

**Figure 5:**
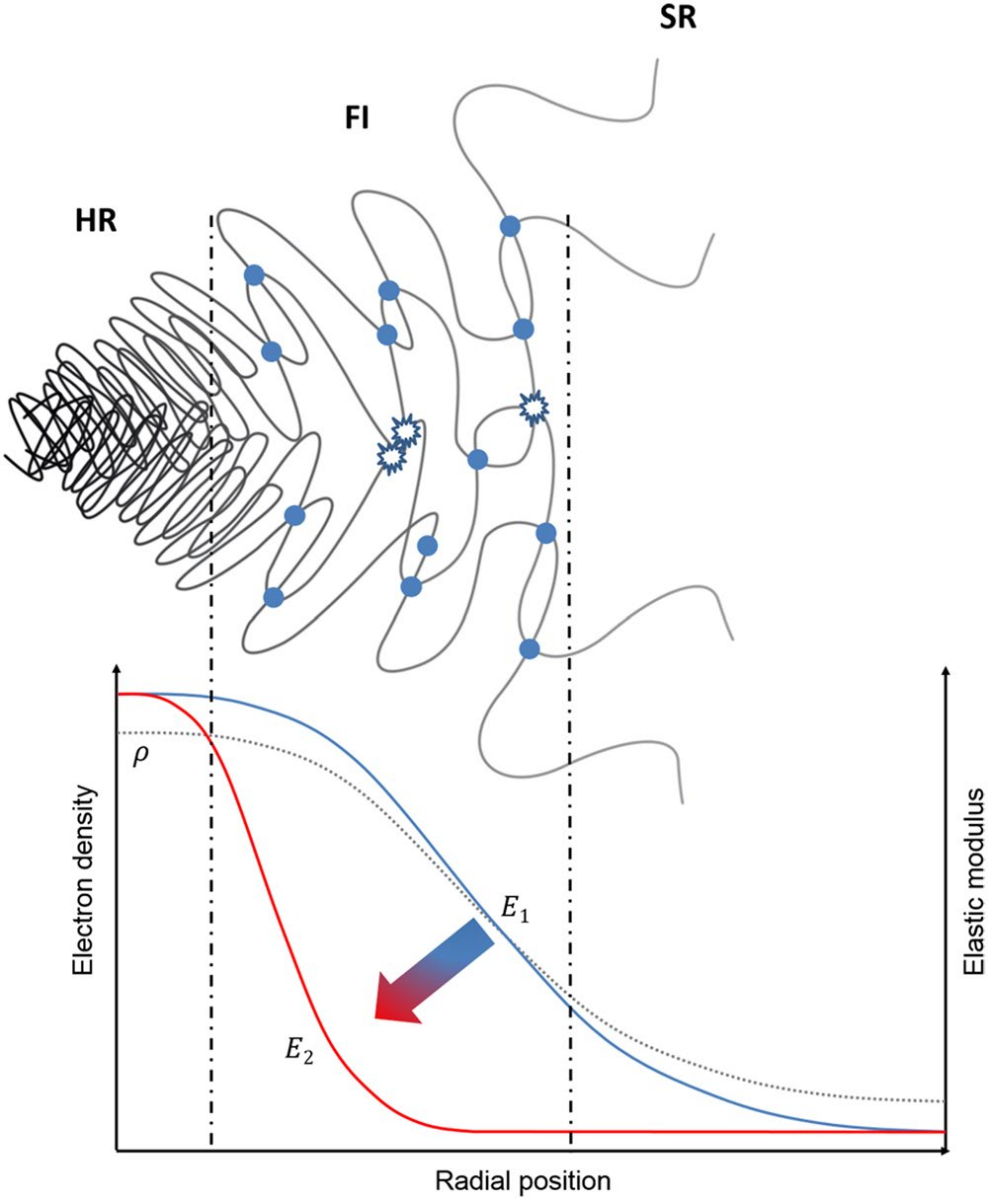
Schematic diagram showing softening mechanism of fuzzy interface. The overall softening process causes gradual destruction of the physical cross-links across the fuzzy interface zone, where the electron density drops steeply from the HR to SR value. This leads to a strong variation of material stiffness as a function of deformation in the fuzzy interface region (FI), whereas the stiffness values in the HR and SR remain largely unchanged.

A schematic diagram of the fuzzy interface softening mechanism within the TPU is shown in Fig. 5. The sketch at the top illustrates the structural transition from the regularly folded, strongly cross-linked hard region (HR) on the left to the low density amorphous soft region (SR) on the right. The fuzzy interface (FI) lying between them is characterized by the gradual change of electron density that approaches that of HR and SR in the corresponding extremes. This is illustrated by the divergent molecular chains and the steeply falling electron density curve in this region. Furthermore, the density of physical cross-links between polymer chains marked by the blue circles also decreases. The combination of these two effects (decrease in density *and* decrease in the density of cross-links) leads to the falling Young’s modulus profile shown in blue. Moderate tensile straining leads to the reduction of the FI stiffness illustrated by the transition from the blue to the red curve in the diagram. Even though the change in electron density (and material density) is insignificant within the FI, the principal mechanism responsible for the change is associated with the loss of physical cross-linking, as illustrated in the Figure. This leads to an even steeper stiffness variation across the FI, and a striking reduction of the overall Young’s modulus of this phase that is ultimately manifested in the Mullins effect macroscopically.

In summary, a description of the Mullins effect was proposed that incorporates multi-phase and hierarchical structure considerations in TPUs. The model was built and refined based on the insights obtained from probing TPU deformation by *in situ* reciprocal space (SAXS/WAXS) analysis under repeated uniaxial tensile loading. The relationships between molecular assemblies at different structural levels and the mechanical properties of the corresponding phases, as well as the length-scale dependent strain evolution were captured by the double-inclusion Eshelby model that was refined by systematic matching of experimental observations. It is concluded that an essential function of the fuzzy interface is to accommodate the strain gradient between hard and soft phases. However, fuzzy interface is the first to undergo tension-induced softening, leading to the appearance of Mullins effect in structural thermo-plastic polymers already at moderate strains. The findings shed light on the molecular function of TPUs, and open the way towards improved design and extended functionality for future applications.

In conclusion, it is worth mentioning that the relevance and importance of nano-scale structural transitions for deformation behavior of materials and composites extends well beyond the confines of the specific material system studied here. The challenge that arises in each specific case concerns the choice of analytical tools that can be successful at obtaining quantitative insights into strain distributions at the appropriate scales. However, the fundamental conceptual and modelling approaches presented in this study possess general relevance, and will be useful in the study of other materials.

## Methods

### Sample

The model TPU system used in the present study was synthesized in the IMC laboratory, Iasi, Romania. The three precursor components were (1) a diisocyanate – 4,4′-dibenzyl diisocyanate (DBDI); (2) a soft segment (SS) macrodiol – polyethylene adipate (PEA); and (3) a small molecule diol as chain extender – ethylene glycol (EG). These were mixed in the molar proportions DI (DBDI): MD (PEA): CE (EG) = 4:1:3, giving hard segment mass fraction of ~40%, and isocyanic index I = 100, meaning that the material was truly thermoplastic. The reaction between DI and MD components was conducted first by vigorous mixing under vacuum at 100 °C, to give a pre-polymer consisting of a macrodiol terminated at each end by diisocyanate. The result was then thoroughly mixed with CE at 90 °C. The polymer was cast into a closed sheet mould and cured at 110 °C for 24 hours, resulting in the production of a sheet with the thickness ~1.0 mm. The detailed pre-polymer synthesis route was described in detail in previous published work by Prisacariu *et al*.^28, 29^. A dog-bone sample, designated “PU185H” with the cross-sectional dimensions of 1.20 × 1.91mm^2^ and a grip-to-grip length of 1.49 mm was prepared from the sheet after at least one-month storage at room temperature, and then used for the tension experiment.

### *In situ* scattering measurements

*In situ* experiment was performed on the B16 beamline at Diamond Light Source (DLS, UK). A Multi-Layer Monochromator (MLM) was used to select the beam energy of 16.5 keV, preserving high X-ray flux for scattering analysis in the SAXS and WAXS regimes. The incident beam was collimated down to 0.2 mm × 0.2 mm spot size and directed at the sample. Scattering patterns were collected in transmission mode in the plane perpendicular to the incident beam direction and parallel to the horizontal loading direction. “X-ray Eye” transmission imaging detector (sCMOS camera, Photonic Science Ltd. UK) was placed in the beam with wide open slits to perform radiography and help identify the region of interest (ROI) on the sample. *In situ* repeated uniaxial tensile loading was carried out at the displacement rate of 1.5 mm/min by a portable mechanical rig (Microtest, Deben Ltd, Suffolk, UK) with a 200 N calibrated load cell. The crosshead displacement and force were recorded and exported from the software for further data processing to evaluate sample extension, strain, stress, etc. At each consecutive loading increment, the imaging detector was translated sideways, and the WAXS detector (Image Star 9000, Photonic Science Ltd., UK) was translated into the beam behind the sample and scattering patterns were collected at the sample-to-detector distance of 178.79 mm. Then the WAXS detector was translated out of the beam to expose the SAXS detector (Pilatus 300 K, Dectris, Baden, Switzerland) located downstream at the sample-to-detector distance of 4545 mm.

In order to determine the sample-to-detector distances precisely, lightly compacted disks of standard silicon powder (NIST SRM 640d) and a disk of Lanthanum hexaboride (LaB6, NIST SRM 660a) powder were placed at the sample position for WAXS calibration. SAXS calibration was obtained from a dry chicken collagen placed in the same position^31, 32^.

### Data interpretation

Macroscopic strain was calculated from the data exported from Microtest software taking into account the specific sample dimensions.

2D SAXS patterns were processed by integrating over a range of azimuthal angles (±20°) straddling the loading direction, resulting in a 1D intensity function *I*_SAXS_ of the scattering vector *q*. Subsequently, 1D plots were fitted with Gaussian curves to determine the center position of the distinct peak *q* ^*^ which was then converted to the structural dimension (*d*) in real space using the relationship *d* = 2π/*q* ^*^. The structural evolution at the nano-scale was calculated from the variation of *q* ^*^ during repeated mechanical loading. Since the peak position *q* ^*^ is most sensitive to the deformation within the “fuzzy interface”, nano-scale strain (*ε*_*SAXS*_) was obtained by comparing the value of *d* at each loading increment with the strain-free reference value.

1D radial binning of 2D WAXS patterns was performed by averaging the intensity across an azimuthal angular range (±20°) over the sector straddling the loading direction. The resultant 1D intensity profile represents the variation of the overall scattering intensity (*I*_*WAXS*_) as a function of *d*-spacing. The degree of crystallinity was calculated as the ratio of the integrated intensity associated with crystal diffraction (*I*_*c*_) to the overall intensity (*I*_*WAXS*_) comprised of the amorphous scattering component (*I*_*a*_) and *I*_*c*_. The amorphous scattering component *I*_*a*_ was fitted with a combination of a Gaussian curve with linear background (*I*_*lb*_). This allowed clear identification and precise analysis of the crystalline peaks (peak 1, peak 2, peak 3 and peak 4) of the *I*_*c*_ curve. These were fitted as Gaussian peaks to determine their center positions. Lattice strains (*ε*_*WAXS*_) derived from the changes in the interplanar *d*-spacing were deduced for each crystalline peak from the shift of the peak center position.

### Double-inclusion Eshelby model

A double-inclusion (two-level) composite model was established that is depicted in Fig. 4. The model quantifies the relationship between the macroscopic applied stress and strains in different components. In particular, it makes it possible to characterize the apparent modulus and elastic modulus of each phase in the TPU, and observe how they change with repeated loading. At level 1 of the model, the inclusions represent the combination of the hard region (HR) with the fuzzy interface (FI) around it, whilst the soft region (SR) serves as a matrix. The stress/strain distribution is written as

1 $${{\rm{\sigma }}}_{m}={f}_{1}{\sigma }_{1}+(1-{f}_{1}){\sigma }_{sr}\,$$

2 $${{\rm{\varepsilon }}}_{m}={f}_{1}{\varepsilon }_{1}+(1-{f}_{1}){\varepsilon }_{sr}\,$$

where (*σ*_*m*_, *ε*_*m*_) denote the *macro scale* stress and strain; (*σ*_1_, *ε*_1_) denote the stress and strain in *inclusion 1* (HR + FI); (*σ*_*sr*_, *ε*_*sr*_) denote the stress and strain in *matrix 1* (SR); and *f*_1_ denotes the volume fraction of the inclusion given by the sum of *f*_*hr*_ (HR) and *f*_*fi*_ (FI).

At level 2, the inclusion represents only the hard region (HR), while the fuzzy interface (FI) serves as the matrix. The stress-strain relationship then changes to

3 $${{\rm{\sigma }}}_{1}={f}_{2}{\sigma }_{hr}+(1-{f}_{2}){\sigma }_{fi}\,$$

4 $${{\rm{\varepsilon }}}_{1}={f}_{2}{\varepsilon }_{hr}+(1-{f}_{2}){\varepsilon }_{fi}\,$$

where (*σ*_*hr*_, *ε*_*hr*_) denote the stress and strain from *inclusion 2* (HR), and (*σ*_*fi*_, *ε*_*fi*_) denote the stress and strain from *matrix 2* (FI); and *f*_2_ is the volume fraction of the inclusion, i.e. HR at level 2.

The relationship between the inclusion strain (ε_*inclusion*_) and overall stress at each level (*σ*_*external*_) can be derived in terms of Eshelby model^33^:

5 $$\begin{array}{c}{\varepsilon }_{inclusion}={K}_{inclusion}^{-1}{\sigma }_{external}=\\ =\{{\{{(1-{E}_{matrix}^{-1}{E}_{inclusion})}^{-1}{[S-f(S-1)]}^{-1}-1\}}^{-1}+1\}{E}_{matrix}^{-1}{\sigma }_{external}\end{array}$$

where *E*_*matrix*_ is Young’s modulus of the matrix, *E*_*inclusion*_ is Young’s modulus of the inclusion, *S* is Eshelby tensor (which can be reduced to a number when the inclusion is assumed to have spherical shape) and *K*_*inclusion*_ is the apparent modulus of the inclusion. This framework allows forward calculation of the composite response to remote loading, and the evaluation of the apparent moduli of the inclusion and matrix at each level. In the present study the model parameters are adjusted to match the observations from the unloading stage of each loading cycle, allowing the moduli of each phase (HR, FI and SR) to be dtermined.

### Data Availability

Additional data can be accessed via ORA (http://ora.ouls.ox.ac.uk). Request for any material samples or specimens described in this manuscript should be directed to the corresponding author.
